# Perceived Support Needs of School-Aged Young People on the Autism Spectrum and Their Caregivers

**DOI:** 10.3390/ijerph192315605

**Published:** 2022-11-24

**Authors:** Kiah Evans, Andrew J. O. Whitehouse, Emily D’Arcy, Maya Hayden-Evans, Kerry Wallace, Rebecca Kuzminski, Rebecca Thorpe, Sonya Girdler, Benjamin Milbourn, Sven Bölte, Angela Chamberlain

**Affiliations:** 1 Telethon Kids Institute, University of Western Australia, Perth 6009, Australia; 2 School of Allied Health, University of Western Australia, Perth 6009, Australia; 3 Autism CRC, Long Pocket, Brisbane 4068, Australia; 4 Curtin Autism Research Group and School of Allied Health, Curtin University, Perth 6102, Australia; 5 School of Psychological Science, University of Western Australia, Perth 6009, Australia; 6 Karolinska Institutet Center of Neurodevelopmental Disorders (KIND), Centre for Psychiatry Research, Department of Women’s and Children’s Health, Karolinska Institutet & Stockholm Health Care Services, Region Stockholm, 171 77 Stockholm, Sweden; 7 Child and Adolescent Psychiatry, Stockholm Health Care Services, Region Stockholm, 104 31 Stockholm, Sweden

**Keywords:** Autism Spectrum Disorder, assessment, diagnosis, goal setting, intervention planning, support needs

## Abstract

With increasing demands for health, disability and education services, innovative approaches can help distribute limited resources according to need. Despite an increased focus on support needs within the clinical pathway and policy landscape, the body of research knowledge on this topic is at a relatively early stage. However, there appears to be a sense of unmet support needs and dissatisfaction with the provision of required support following an autism diagnosis amongst caregivers of young people on the spectrum. The primary aim of this study was to explore the perceived support needs of Australian school-aged young people on the spectrum and their caregiver(s). This was achieved using a phenomenographic Support Needs Interview conducted by occupational therapists during home-visits with caregivers of 68 young people on the spectrum (5–17 years). Qualitative data analysis resulted in two hierarchical outcome spaces, one each for young people and their caregivers, indicating interacting levels of support need areas that could be addressed through a combination of suggested supports. These support needs and suggested supports align with almost all chapters within the Body Functions, Activities and Participation and Environmental Factors domains of the International Classification of Functioning, Disability and Health. The overall goals of meeting these complex and interacting support needs were for the young people to optimize their functioning to reach their potential and for caregivers to ensure the sustainability of their caregiving capacity. A series of recommendations for support services, researchers and policy makers have been made to position support needs as central during the assessment, support and evaluation phases.

## 1. Introduction

The prevalence of autism diagnoses among children and adolescents (referred collectively hereinafter as young people) has increased substantially over recent decades, in part driven by a greater awareness of the condition and the need for formal diagnosis to access support [1,2]. With an increasing number of families seeking professional support, wait times for services have become problematic. This is underpinned by insufficient workforce resources to meet the demand, particularly in regional and remote locations [3]. In addition, the ‘out-of-pocket’ costs to families and government/non-government expenditure in providing support for young people on the autism spectrum have increased dramatically [4]. Subsequently, the existing health, disability and education systems that have traditionally provided these supports are unable to balance goal attainment and sustainability, calling into question their appropriateness. Innovative approaches are needed to determine necessary supports within complex systems and provide them in a timely and cost-effective manner, in a way that enables individuals to achieve their most important personally meaningful goals and maximize their quality of life [5].

### 1.1. Local and International Context in Determining Necessary Supports

Globally, there has been a societal shift towards focusing health, disability and education supports to go beyond decreasing symptoms and dependence and towards optimizing participation and engagement in important life situations [6,7,8]. Arguably, the most influential conceptual framework introduced to assist interprofessional stakeholders across sectors to make this transition is the International Classification of Functioning, Disability and Health (ICF [7]). This bio-psycho-social model recognizes that there is a continuum between states of disability (encompassing impairments, activity limitations and participation restrictions) and functioning (encompassing body functions, activities and participation), and that these states are influenced by health conditions and contextual factors (personal and environmental). The ICF has a cascading structure where increasing detail is included to classify concepts across four domains. At the first level of classification, the Body Structures and Body Functions domains have eight chapters each, the Activities and Participation domain has nine chapters and the Environmental Factors domain has five chapters [7]. The comprehensiveness of the ICF is a strength; however, the 1600+ codes present implementation challenges in practice. To address this barrier, shortlists of relevant ICF codes have been produced for specific health conditions using a rigorous research process. This has included the development of the ICF Core Sets for Autism [9], encompassing the Brief ICF Core Set for school-age children and adolescents 6–16 years (referred to hereinafter as the Brief ICF Core Set). This Brief ICF Core Set includes 81 codes, spanning 16 of the 30 chapters in the ICF, that are considered most relevant for young people on the spectrum.

Paralleling this conceptual shift, there has been an international policy shift towards individualized funding [10,11,12]. Within the Australian context in which this study was conducted, the National Disability Insurance Scheme (NDIS [10]) (p. 23) was introduced with guiding principles to facilitate people with a disability in accessing reasonable and necessary supports. The administration of individualized funding, such as the NDIS, presents complex challenges to consumers, support services and funders [5]. Whilst symptom severity, impaired functioning and increased support needs are to some extent associated with each other [13,14], a support needs measure, as opposed to an adaptive behavior measure (a sub-set of functioning measures), is better able to clearly determine the support needs required [15]. Hence, a paradigm shift towards a model of service that positions support needs as central in service planning, has the potential to, at least in part, solve the real-world problem facing the autism community and its support services.

One practical step that has occurred towards this solution is the inclusion of a comprehensive needs assessment as the initial phase within Australia’s national guideline for the assessment and diagnosis of autism [16]. The comprehensive needs assessment consists of both a medical evaluation and an assessment of functioning, with the joint purpose of exploring the “*key strengths, challenges and needs that inform future clinical management and service delivery*” (p. 11). The assessment of functioning can be conducted by specified health professionals in collaboration with the individual on the spectrum and their caregiver(s)/support people. Topics for inclusion include functioning across a broad range of domains, personal strengths, environmental factors and support needs. Information should be collected through a combination of file review, interview, observation, standardized measures and/or interprofessional communication. The expected outcomes of an assessment of functioning are “*the identification and prioritisation of observed and expressed support needs*” and “*connection to appropriate services based on the client’s support needs where impaired functioning is identified*” [16] (pp. 31–32).

### 1.2. Support Needs as a Foundation for Determining Necessary Supports

Based on dictionary definitions, the term ‘support needs’ can be interpreted as follows: “*to require (the provision or availability of services)*” that are “*essential or very important (rather than merely desirable)*” in order to “*enable (someone) to fulfil its function*” or “*contribut(e) to the success of something*” (compiled from the entries for ‘support’ and ‘need’, [17]). In the context of intellectual and developmental disabilities, support needs have been defined as “*a psychological construct referring to the pattern and intensity of supports necessary for a person to participate in activities linked with normative human functioning*” within an individual’s personal and environmental context [18] (p. 135). Traditionally, support needs have been conceptualized based on the type, time, intensity and importance of support required over a range of health and functioning domains [13,19,20,21,22]. Each support needs measure focuses on different (and at times overlapping) concepts, with most designed for use with adults who have a specific disability [13].

A sense of unmet support needs and dissatisfaction with the provision of required supports following an autism diagnosis is a significant issue amongst caregivers of young people on the spectrum, compounded by the presence of more complex support needs and progression towards adulthood [23,24,25,26,27,28]. Caregivers of young people on the spectrum report multiple high priority support needs, that vary according to the age of their care recipient [22,23,28,29]. Specific support services that caregivers prioritized, but were not always able to access, included activity-based programs, childcare/out-of-school-care, early intervention (intensive behavioral and other), learning support at school, therapy sessions (to address challenges with behavior, communication, diet, independence, mental health, sensory and/or sleep), life skills training, medication, respite care, social skills groups and vocational rehabilitation [22,23,24,28,30]. Other important core support services were required to meet needs for increased knowledge about autism, effective strategies to utilize at home, awareness of available support services, coordination between members of the interprofessional team, connection with other caregivers of other young people on the spectrum, caregiver mental health, housing and greater societal acceptance in relation to autism [22,28,29,30,31]. Finally, caregivers often report other support needs that are met through informal systems, related to opportunities for all family members, including the young person, to build and maintain their health, capacity and social connectedness [22,30].

Caregivers with lower health literacy, along with those from culturally and/or linguistically diverse backgrounds, often experience greater informational support needs, alternate preferences in relation to the provision of support, additional barriers to accessing required supports and/or greater dissatisfaction [28,31,32]. Further, caregivers with less financial resources have greater unmet support needs, with additional barriers including a lack of funding for support services, for example, from government or non-government systems [22,23,24]. Other contributors to unmet support needs include service availability, wait times, the young person’s fear of professionals, uncertainty surrounding required supports, congruence with family schedules and the absence of an informal support system [24,28,30]. These contributors are likely to vary across countries due to differing societal attitudes, services, systems and policies.

### 1.3. Research Aim and Objectives

Given the relatively early stage of research into support needs of young people on the spectrum, a qualitative approach was selected as the most appropriate methodology to ascertain the breadth of experiences within a single context. The primary aim of this study was to explore the perceived support needs of Australian school-aged young people on the spectrum and their caregiver(s). Specific objectives were to: (1) describe the most important support needs from the perspective of their caregivers; (2) investigate the breadth and frequency of body function impairments, activity limitations, participation restrictions and environmental barriers underlying these support needs; and (3) investigate the breadth and frequency of past, current and/or potential environmental supports that were perceived as likely to address these support needs.

## 2. Materials and Methods

### 2.1. Research Design

Phenomenographic Support Needs Interviews were chosen to explore the relationship between the subjective (inner world of the caregiver) and objective (activities involved in supporting a young person on the spectrum within their usual contexts) as the central phenomenon [33]. This approach directs the researcher in reflecting on the various descriptions gathered from the participants, forming a more holistic awareness of the phenomenon and its meaning [33,34], delivering a second order perspective where the phenomenon “*is described as it is understood rather than as it is*” [33] (p. 1425). This study was undertaken in the context of a larger research program on Reliability, Validity and Usability of Assessment of Functioning Tools for Autism and Neurodevelopmental Conditions in the Australian Context.

### 2.2. Participants and Recruitment

Participants were eligible to participate in the larger research program if they were an unpaid caregiver of an individual under 21 years of age who had been diagnosed with a neurodevelopmental condition, was a NDIS participant, and lived in one of four states in Australia (New South Wales, Queensland, Victoria or Western Australia). Interpreter services were available, hence no language exclusion criteria were applied. The recruitment strategy was multifaceted, involving the agency implementing the NDIS forwarding an invitation letter to 2800 eligible caregivers and social media/database promotion by the research team and Australian services. This resulted in a convenience sample of 174 caregivers who received a participant payment in appreciation for their time and inconvenience (AUD 50-200). Participants for this study were selected from the larger sample if the young person had an autism diagnosis (inclusive of current and previous diagnostic labels), was of school age in their state (regardless of attendance status), and had available data for the Support Needs Interview.

### 2.3. Data Collection

The data collection protocol (Supplementary File S1) was co-designed by a team of occupational therapy (OT) researchers and caregivers of young people on the spectrum (including two neurodiverse individuals), with input from a multidisciplinary team (pediatrician, psychiatrist, psychologist and speech pathologist). The data collection protocol was piloted by OTs with six caregivers of young people diagnosed with a neurodevelopmental condition, and small refinements made prior to commencing the research project. Data was collected during a home-visit by a registered OT with a research qualification and experience working with young people on the spectrum and their families. The OTs underwent extensive training in the data collection protocol, including administration of relevant measures.

#### 2.3.1. Demographic and Clinical Characteristics

A background survey included questions about the demographic and clinical characteristics of the young person, their caregiver(s) and their family (Supplementary File S1). The caregiver-reported questions were presented online using REDCap [35,36] with fixed-response items (single or multiple response options) and short text boxes, addressing the following:

Young person’s date of birth, school year, gender, autism diagnostic label (with age when assigned) and other diagnoses.

Caregiver’s date of birth, gender, relationship to the young person, along with their self-perceived health measured using the EQ-5D-5L [37] and wellbeing measured using the Personal Wellbeing Index-Adult (PWI-A [38]). The EQ-5D-5L item included in this study is a visual analogue scale (where 0 = the worst health you can imagine and 100 = the best health you can imagine) and the measure has established test–retest reliability and convergent validity [37,39]. The PWI-A has seven items corresponding to wellbeing domains (standard of living, health, achieving in life, relationships, safety, community-connectedness and future security) that are scored on an 11-point scale (where 0 = No satisfaction at all and 10 = Completely satisfied) and averaged to produce an overall wellbeing score [38]. The PWI-A has established internal consistency, test–retest reliability, construct validity, convergent validity and normative data [38].

Family’s (young person and/or caregiver) history of neurodevelopmental conditions, First Nations status, identification with a specific cultural group, language other than English spoken at home and home postcode.

In order to describe the young person’s clinical characteristics further, two standardized measures were administered by the OTs in their published form and results were subsequently entered into the REDCap database. These were:

Autism Mental Status Exam (AMSE [40]) measures the young person’s autism symptoms and was administered during the home-visit. The AMSE is an 8-item observational measure (supplemented by caregiver-report) scored on a three-point scale (where 0 = Not impaired; 1 = Reported but not observed; and 2 = Impaired). A total score was calculated by adding the items together, resulting in a score between 0–14, where a threshold of ≥5 was used to indicate that symptoms were congruent with an autism diagnosis [40]. The AMSE has established internal consistency, inter-rater reliability, concurrent validity and diagnostic accuracy among young people on the spectrum [40,41].

Vineland Adaptive Behavior Scales, Third Edition (Vineland-3 [42]) measures adaptive and maladaptive behavior and was administered using the Comprehensive Interview Form during a telephone call within approximately one week of the home-visit. This measure contains an item pool of 502 questions spanning five domains (communication, daily living skills, socialization, motor skills and maladaptive behavior). The items administered were determined through the online Q Global platform (Pearson Inc., San Antonio, TX, USA) based on basal and ceiling rules, and were scored on a three-point scale (where 0 = Does not; 1 = Sometimes; and 2 = Usually/regularly in reference to the frequency that behavior is present). The adaptive behavior composite and domain standard scores, along with the maladaptive v-scale scores, were calculated by Q Global and were interpreted according to the categories outlined in the manual where scores within two standard deviations of the mean were considered normal [42]. The Vineland-3 has established internal consistency, test–retest reliability, inter-rater reliability, content validity, construct validity, criterion validity and overall utility [42,43,44].

#### 2.3.2. Support Needs Interviews

Existing standardized measures aiming to identify support needs related to young people on the spectrum were investigated (e.g., [13,22]), and deemed unsuitable due to the restricted response options, limited age ranges, focus on other conditions, training requirements and/or administration time. Subsequently, a semi-structured Support Needs Interview (Supplementary File S1) was designed for face-to-face administration during a home-visit [16]. Caregivers were initially asked to identify the five to ten most important support need areas related to the young person within their care, including new (unmet) or existing (partially or fully met) support needs. Caregivers were then encouraged to describe their rationale for the support need, along with existing supports and new supports required. The Support Needs Interview allowed the researcher to prompt the caregiver about support needs raised earlier in the home-visit and suggest new supports that had not been considered. Finally, the caregiver was asked to rank the support needs from most to least important. The Support Needs Interview was audio recorded to aid recall for the researcher when creating an ‘interview summary’ in the REDCap database, which was primarily documented in the OT’s words (although direct quotes from the caregiver were occasionally included). This interview summary included a short name and description (unmet need, existing supports, suggested new supports) for each support need. After the home-visit, the OT prepared a written report for the caregiver that included the Support Needs Interview findings. Caregivers were invited to provide feedback about this report during a video conference meeting or an online survey, as a form of member checking (Supplementary File S1).

### 2.4. Data Analysis

Vineland-3 data that were transferred into REDCap from the Q Global generated report format were checked, and if applicable edited, by a second researcher to ensure 100% accuracy. New variables to describe the family’s geographical location [45] and socio-economic status (Index of Relative Socio-Economic Advantage and Disadvantage, IRSAD, [46]) were assigned using the postcode. Data was exported from REDCap to SPSS [47], where composite scores were computed and age was calculated based on dates of birth and assessment. Data were described using frequencies (*n* for participants, *k* for support needs and % for proportion of total available sample), range, mean (*M*) and standard deviation (*SD*).

The interview summaries were de-identified before importing into NVivo [48] for phenomenographic analysis using the following seven steps [34]:

Familiarization (step 1)—The first and senior author read each of the 68 interview summaries in full. In addition, the researchers who conducted the Support Needs Interviews were provided with an opportunity to review the interview summaries they had written (range = 5–19 per assessor).

Compilation (step 2)—To address the first research objective, the first and senior authors independently coded the short name of each of the top three prioritized support needs to the most applicable ICF chapter if possible [7], referring to the more detailed response if required. These codes were compared (65.20% full and 25.98% partial agreement) and any disagreements were resolved through discussion. To address the second and third research objectives, the first author then deductively coded the full interview summary for each participant against a coding framework that included: interview question, recipient(s) of the support, the extent that the support need was currently being met, priority ranking and relevant ICF chapters [7]. The senior author reviewed the coding to confirm that the ICF chapters were represented in the interview summary (98.69% agreement). Where coding was not confirmed, the first and senior author discussed the responses to reach consensus on the number of cases assigned to each chapter.

Condensation (step 3)—The first author systematically reviewed the responses coded to each ICF chapter endorsed by at least one-quarter (*n* ≥ 17) of caregivers (categories). A combination of deductive and inductive coding was used to create meaningful concepts within each category, using ICF two-level classifications to guide this process. The length of coded responses was reduced during this process to focus on the central aspects of each concept.

Grouping (step 4)—The first author collated responses for each concept and organized them either separately or hierarchically within each category. They were organized separately for young people compared to caregiver(s) and others, and described in a summary paragraph. The six researchers who conducted the Support Needs Interviews were invited to review the summary paragraphs and provide feedback on emerging findings.

Preliminary comparison (step 5)—The first author reflected on the researchers’ feedback (*n* = 4) on the summary paragraphs for each category using a high-level view, where the boundaries between categories were considered. Although the initial deductive coding against ICF chapters resulted in relatively consistent boundaries, this process resulted in two categories being divided into two further categories (relating to the ICF chapters *Interpersonal interactions and relationships* for young people and *Services, systems and policies* for caregiver(s) and others).

Naming (step 6)—In recognition that the ICF was used as a conceptual model for understanding support needs in this study, the first author named categories according to the relevant ICF chapter. In addition, a brief phrase capturing the key concepts within each category was articulated and the summary paragraphs for each category were revised.

Contrastive comparison (step 7)—The first author visually arranged the category names (with the key concepts) to explore the similarities and relationships between categories. This led to the creation of two outcome spaces, where the categories were represented as hierarchical figures for young people and caregiver(s) and others. There was a common thematic framework across both outcome spaces, where a range of difficulties with body functions and activities and participation were identified as primary, secondary or tertiary support needs. Caregivers reported that these support needs could be addressed through the provision of environmental supports, leading to a positive outcome. The six researchers who conducted the Support Needs Interviews and senior author were invited to review and provide feedback on the findings again at this point and their feedback was integrated into the final findings section that was shared with the other authors for review.

Trustworthiness of the phenomenographic analysis was achieved using strategies to enhance credibility, transferability, dependability and confirmability [34,49]. Credibility was enhanced through the large multi-site sample leading to varying perspectives of support needs; timing of the Support Needs Interview to allow triangulation with other information collected during the research study; opportunity for caregivers to engage in member checking; expertise of the researchers who administered the Support Needs Interview; and peer review of the findings by multiple researchers (including inter-rater counts for frequencies). Transferability was enhanced through detailed descriptions of the young people, caregivers, families, support needs and suggested supports. Dependability was enhanced through the detailed descriptions of the materials and methods, along with peer review of the findings (where consensus was reached for frequencies). Confirmability was enhanced through the large sample; focus on categories endorsed by at least one-quarter of caregivers; triangulation with the ICF as a theoretical perspective; qualitative research experience of the authors; and use of reflexive analysis to maintain awareness of the potential impact of the researchers’ professional and personal experiences on the findings.

## 3. Findings

### 3.1. Participants

The 70 caregivers who participated in this study reported on 68 school-aged young people on the spectrum (one caregiver reported on two siblings) (Table 1). The young people were aged between 5 and 17 years (*M* = 10.82, *SD* = 3.09), with two-thirds in primary school (*n* = 45, 66.17%) and there were slightly more males (*n* = 42, 61.76%) than females (*n* = 26, 38.24%). Autism Spectrum Disorder was the most common autism diagnostic label (*n* = 57, 83.82%), with all severity levels represented. The average age of initial autism diagnosis was 5.70 years (*SD* = 2.94), with AMSE results [40] confirming that most young people had autistic signs and symptoms above the clinical threshold (*M* = 5.12, *SD* = 2.39) and more than half had at least one co-occurring neurodevelopmental condition (*n* = 39, 57.35%). Vineland-3 results [42] revealed low levels of adaptive behavior composite scores (*M* = 65.55, *SD* = 14.76) and moderate to high maladaptive behavior scores (Internalizing and Externalizing: *M* = 21.55 and 19.36, *SD* = 1.61 and 2.50, respectively). The caregivers (Table 1) ranged between 29 and 67 years of age (*M* = 42.77, *SD* = 7.52) and were almost exclusively mothers (with the exception of three fathers and one grandmother). EQ-5D-5L visual analogue scores [37,50] indicated the caregivers were in the below average range of self-perceived health (*M* = 67.81, *SD* = 19.39) and the PWI composite wellbeing score [38,51] was also below the average range (*M* = 59.01, *SD* = 22.73). Over half of the families (Table 1) had a history of neurodevelopmental conditions (*n* = 31, 53.45%), approximately one-quarter of the families reported cultural and/or linguistic diversity (*n* = 19, 27.94%) and the families predominantly lived [45,46] in metropolitan locations (*n* = 60, 89.55%) associated with moderate socio-economic status (IRSAD decile: *M* = 6.87, *SD* = 2.62).

### 3.2. Overview of Support Needs

**Figure 1:**
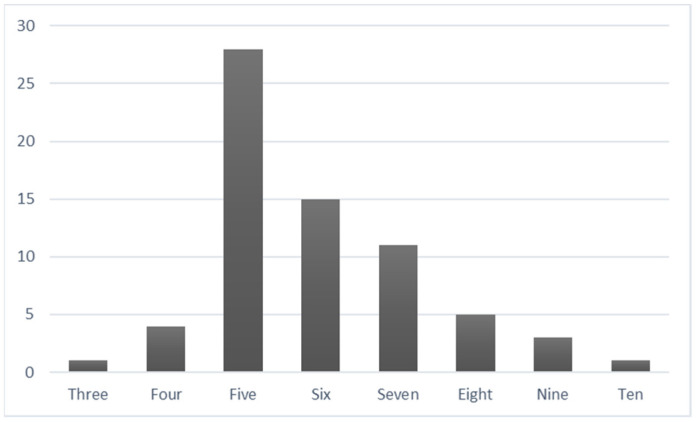
Frequency of young people (*n* = 1–28) associated with each number of total reported support needs (*k* = 3–10).

During the Support Needs Interviews, the caregivers described a total of 403 support needs, averaging approximately six support needs each (*M* = 5.93, *SD* = 1.37, range = 3–10, Figure 1). All caregivers described support needs where the young person was the recipient, two-thirds of the caregivers described themselves as being the recipient of at least one support need (*n* = 46, 67.65%) and two-fifths describe another recipient for at least one support need (*n* = 28, 41.18%; e.g., sibling, wider family unit, educator, support worker). Half of the caregivers reported at least one support need that was currently being met with a need to maintain the supports (*n* = 34, 50.00%). Almost all caregivers reported that at least one support need was being partially met and further support was required (*n* = 65, 95.59%). Further support included: increasing the frequency and/or duration of an existing support; expanding the scope of an existing support to address additional support needs; introducing a new support to supplement a support already in place; changing the support provider or location to better meet a support need; prescribing a product (e.g., medication, equipment) or change to the environment; and facilitating informal supports. Finally, two-fifths of the caregivers described at least one support need that was not currently being addressed at all (*n* = 28, 41.18%), although in a small proportion of cases there were plans in progress to initiate or re-establish previous supports.

In terms of the first research objective, the main underlining difficulty for the top three prioritized support needs was most frequently related to multiple ICF chapters (*k* = 66, 32.35%), where this either spanned a combination of chapters from the Body Functions and Activities and Participation domains (*k* = 37, 18.14%) or multiple chapters from the Activities and Participation domain alone (*k* = 29, 14.22%). Where the difficulty underlying the support need could be attributed to a single ICF chapter, this was most frequently Mental functions (*k* = 43, 21.08%), Major life areas (*k* = 25, 12.25%), Interpersonal interactions or relationships (*k* = 24, 11.76%).

In relation to the second and third research objectives, analysis of the full data set revealed that the support needs and/or suggested supports spanned three domains of the ICF (Body Functions, Activities and Participation and Environmental Factors). All but two caregivers (*n* = 66, 97.06%) described at least one support need related to Body Functions and all caregivers described at least one support need related to both Activities and Participation and Environmental Factors (Table 2). At least one-quarter (*n* ≥ 17) of caregivers described support needs associated with three of the eight Body Functions chapters, all nine of the Activities and Participation chapters and three of the five Environmental Factors chapters. Support needs and suggested supports that were mentioned by at least one-quarter of the participants (*n* ≥ 17) are presented in the following outcome spaces and elucidated in the following sections, presented from the most to least frequent for young people and caregiver(s)/others.

### 3.3. Young People

**Figure 2:**
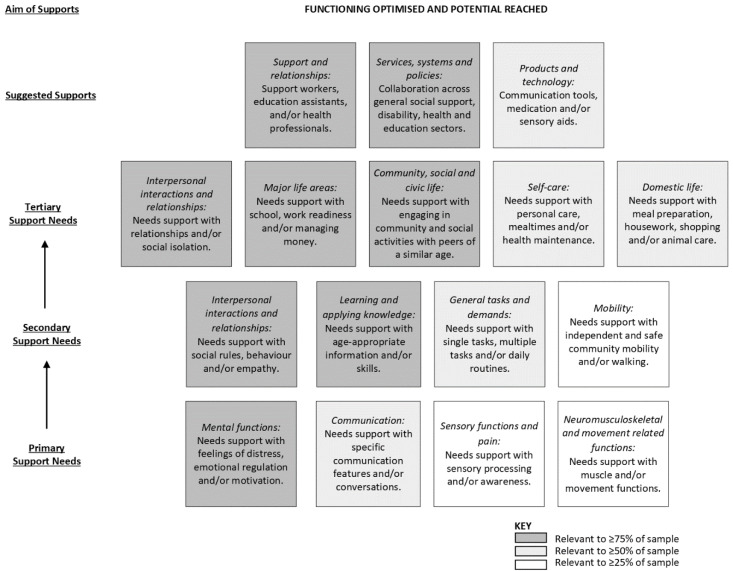
Hierarchical outcome space for young people, illustrating three interacting levels of support needs (primary to tertiary) that can be addressed through a combination of three types of suggested supports to achieve the overall aim for the young person.

The support needs of young people involved a hierarchical outcome space of three interacting levels of support need areas that could be addressed through a combination of three types of supports (Figure 2). The aim of meeting these support needs was to prepare the young person to function at an optimal level so they could reach their potential, which was associated with enhanced mental and physical health; engagement and endurance in activities and roles; belonging and social connectedness; and readiness to transition to independent living as an adult. This was explained by a mother of a 16-year-old daughter on the spectrum as:

“*being about (their) ability to play (their) role in society and to feel included. This includes support to transition into employment (and) possibly other roles as (they) become an adult, but also (their) genuine inclusion (not tokenistic inclusion) and (their) ability to be involved in … communities and any other hobbies that (they have) an interest in*”.

#### 3.3.1. Exploration of Primary Support Needs

Mental functions—Many young people experienced difficulties with feeling distressed, emotion regulation and motivation. Feeling distressed encompassed worrying, becoming overwhelmed, psychological inflexibility (especially in relation to rules, routines and/or unfamiliar environments), responses to fear inducing events (e.g., medical appointments) and diagnosed anxiety disorders. Emotional regulation became challenging when feelings of distress escalated, resulting in challenges in calming down, reacting in a socially acceptable manner, understanding the consequences of their actions and responding to the emotions of others. These emotional regulation difficulties often presented as crying, screaming, meltdowns, shutting down, self-injurious behaviors or physical violence. Motivation, or the lack of, was described as a critical component that determined if the young person would maintain attention and engage in an activity.

Communication—Some caregivers described the young person’s communication difficulties more broadly, while others described discrete concerns with receiving and producing messages. Challenges related to receiving messages included language processing (especially multi-step instructions), understanding verbal and non-verbal social cues, interpreting implied meaning and tolerating specific words (e.g., “*No*” needs to be replaced by “*Maybe later*” or “*You need to wait*”). Challenges with producing messages included pronouncing some sounds/words, stuttering, communicating in sentences, displaying appropriate affect and expressing needs, preferences and feelings. Other difficulties included initiating and maintaining conversations that involve reciprocity (e.g., turn taking, rather than focusing on their own interests), flexibility (e.g., ability to discuss a variety of topics or continue a conversation if it does not follow a practiced script) and appropriate language (e.g., recognizing when a word might be offensive).

Sensory functions and pain—Some young people experienced hyper- and/or hypo-reactivity to sensory input, including pain, environmental safety cues, textures, noise, touch, movement, proprioception and/or spatial awareness.

Neuromusculoskeletal and movement related functions—Some young people experienced reduced strength/tone (especially core and hand muscles), which negatively impacted balance, joint stability and postural control. Difficulties with coordination, fine and gross motor skills and/or motor planning were also described.

#### 3.3.2. Exploration of Secondary Support Needs

Interpersonal interactions and relationships—Many young people were described as having limited social skills. Specifically, they had difficulty learning and applying social rules and demonstrating socially appropriate behavior, such as being honest, judging if language/topic is offensive, maintaining personal space, regulating emotions, hyper-focusing on individuals, navigating disagreements and sharing attention between all parties involved in the interaction. They also had difficulties understanding and responding empathetically to diverse perspectives, feelings, interests and needs of others.

Learning and applying knowledge—The young people experienced difficulties with learning age-appropriate information and skills, impacted by insufficient confidence, co-occurring conditions (e.g., dyscalculia, dyslexia, dysgraphia) and/or other processing difficulties. Challenges applying knowledge were also reported, specifically with respect to focusing attention, solving problems and making decisions. For children, this impacted the ability to learn to read, write and calculate. In adolescents, it affected the ability to learn study and research strategies, produce complex written tasks and learn at the same pace as peers. Along with educational restrictions, learning across many other areas of Activities and Participation was impaired.

General tasks and demands—The young people experienced difficulties with undertaking single tasks, multiple tasks and carrying out daily routines. They struggled in focusing their attention, maintaining concentration and following instructions for single tasks, especially within noisy environments. They also found undertaking multiple tasks challenging, due to difficulties with sequencing, making decisions, managing time, solving problems, being flexible, following multi-step instructions and executing plans. This was particularly problematic in relation to daily self-care and school routines, leading to fatigue, lack of motivation and extended time requirements. Situations characterized by change, transitions, inconsistency or unpredictability were distressing and resulted in a logistically challenging routine.

Mobility—The young people experienced difficulties independently and safely navigating their local community using public transportation, bicycles and/or on foot due to an array of barriers, including anxiety, awareness of surroundings, checking for oncoming traffic and money management. Some young people who had underlying impairments in gait, proprioception, balance, coordination and/or joint stability had difficulty walking.

#### 3.3.3. Exploration of Tertiary Support Needs

Interpersonal interactions and relationships—The young people experienced difficulties with friend, peer, family member and health professional relationships. Caregivers expressed concern regarding the young person’s ability to establish and maintain friendships, with many noting that the young person had no friends at all. They were also concerned about limited positive informal relationships, characterized by acceptance and inclusion, with peers of a similar age within school, play and extracurricular environments. While some young people preferred interacting with neurotypical peers, some caregivers felt that relationships with other young people on the spectrum facilitated social connection and age-appropriate social experiences (e.g., playdates and sleep overs), minimizing social isolation. In addition, older adolescents were interested in forming intimate relationships with a romantic partner. Caregivers also described difficulties facilitating cooperative and caring relationships with siblings, and in some families, with parents and extended family members. Finally, young people found it hard to connect with new health professionals, which was frequently encountered due to high staff turnover.

Major life areas—Many young people experienced difficulties with education, associated with academic, behavioral, cognitive, emotional, motivational, sensory, social and/or transition support needs. For older adolescents, challenges related to vocational training (e.g., assignment completion to obtain a TAFE qualification) and transitioning into volunteer roles were reported. Some young people experienced difficulty managing money, including purchasing items in a shop (e.g., due to dyscalculia) and budgeting (both in the short term, such as keeping money aside for the public transport fare for their return trip at the end of an outing, and medium term, such as achieving a savings goals).

Community, social and civic life—The young people experienced difficulties with engaging in community, social and civic life, limiting their opportunities for socialization with peers of a similar age. Young people needed support engaging in play, hobbies, sports and community based informal associations, within a range of home and community activities (Table 3).

Self-care—The young people experienced difficulties with personal care, mealtimes and health maintenance. Personal care activities where full independence was not demonstrated included bathing, washing/brushing hair, brushing teeth, selecting clothes, dressing and toileting (including incontinence for some children). Mealtime challenges were of great concern due to the potential for nutrient deficiencies, and included restricted diet (e.g., associated with certain textures, colors, tastes, smells, preparation methods, combinations, novelty), regulating intake (e.g., under and overeating, including due to body image concerns and anxiety eating in front of others), hoarding food, consuming non-food items and managing cutlery. Health maintenance was associated with numerous concerns, including self-injurious behavior (e.g., biting lip, picking at nail bed, bumping body against surface), suicidal ideation, anxiety about preventative health visits (e.g., GP, dentist), weight (e.g., body image, obesity), vulnerability to bullying, hazard identification and risk-taking behaviors (e.g., absconding, crossing roads, interactions with strangers in-person/online, drugs, illegal activity).

Domestic life—Many young people needed support with age-appropriate domestic life skills, particularly older adolescents transitioning to adulthood and more independent living (either in their own home, on their caregiver’s property or in supported accommodation). This was underpinned by motivation and regulation difficulties, along with a lack of opportunity to develop these skills and minimal preparation for this transition. Caregivers were eager for young people to take on increased responsibility for helping with meal preparation (e.g., making breakfast, preparing a small snack), housework (e.g., tidying their bedroom, disposing of garbage), shopping (e.g., putting groceries away) and animal care (e.g., walking dog).

#### 3.3.4. Suggested Supports to Meet Support Needs

Support and relationships—Identified supports included access to personal care providers and health professionals, in addition to the extensive informal support provided by the caregiver and other members of their immediate family. Personal care providers included knowledgeable and experienced support workers (to assist with applying skills learnt in therapy, community participation, domestic life, homework, self-care routines and/or transport to school, appointments and other community-based activities) and education assistants (to assist with maintaining attention, communication, emotional regulation, following instructions, learning, socialization and/or therapeutic strategies). Health professionals included medical doctors (pediatricians, and occasionally general practitioners or psychiatrists); an array of allied health professionals (e.g., dietitians, OTs, physiotherapists, psychologists and speech pathologists); and interprofessional teams that target specific areas (e.g., behavior management programs, mealtime support, mental health services, music therapy, social skills groups and vocational rehabilitation). In particular, OT and facilitated life skills groups were identified as key supports in developing independence in age-appropriate activities (e.g., public transport, meal preparation, housework, shopping, money management).

Services, systems and policies—The young people needed access to general social support, health and education services and systems. Flexible funding was needed to fund support workers, transport services, group programs (general social support systems) and health professionals (general social support and health systems). Caregivers described a range of short weekly group programs and longer school holiday programs at school or in the community (general social support services), where the ideal cohort was sometimes skill-matched autistic peers and other times neurotypical peers. The focus of the group programs spanned interpersonal skills development (e.g., social skills), recreation (e.g., Lego, art, music, swimming, group sports, social outings) and domestic life (e.g., cooking). In addition to funding for health professionals, there was an identified need for access to health workforces within reasonable wait times (health services). In relation to education services, caregivers described the importance of a positive educational culture that is inclusive of individuals on the spectrum, curriculum that is engaging (e.g., freedom to follow interests, fun tasks, sufficiently challenging, unconventional learning approaches), relevant (e.g., life skills, such as cooking and banking), flexible (e.g., learning programs to meet individual needs, modified activities, regular breaks, therapy services provided at school, at-home therapy activities count as homework), and adaptable (e.g., additional staff for unfamiliar events, handover plan for relief and external staff). This requires sufficient individual and school-based funding to employ education assistants when required and allow educators time to tailor the curriculum and collaborate with other stakeholders to develop and implement individualized education plans. Caregivers also emphasized the support need for collaboration between general social support, health and education systems, with communication channels to ensure young people, caregivers, personal care providers, health professionals, educators and case managers co-operate effectively together to meet support needs.

Products and technology—Supports identified included communication devices, medication and/or sensory aids. Communication tools ranged from printed visual prompts (e.g., checklists, schedules, social stories and color-coded feeling charts with images) to assistive technology (e.g., speech-generating devices, or other electronic devices such as iPads or laptops). Medication was used to address anxiety, attention, depression, sleep difficulties and vitamin deficiencies. Sensory aids included fidget toys, noise cancelling headphones, swings and weighted blankets/clothes.

### 3.4. Caregiver(s) and Others

**Figure 3:**
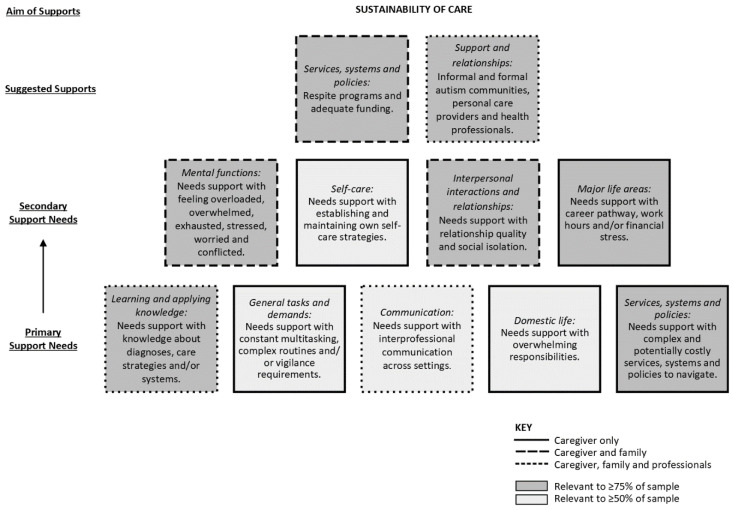
Hierarchical outcome space for caregivers and others, illustrating two interacting levels of support needs (primary and secondary) that can be addressed through a combination of two types of suggested supports to achieve the overall aim for the caregivers and others.

The support needs of primary caregivers and other support people involved a hierarchical outcome space of two interacting levels of support need areas that could be addressed through a combination of two types of supports (Figure 3). The aim of meeting these support needs was to maintain the sustainability of their caring capacity, which was associated with enhanced coping and role balance; mental and physical health; and quality of life. This was illustrated by a mother of three daughters on the spectrum (aged 8, 10 and 12 years):

“*I have little to no support. If I fall to pieces, how am I going to support my kids? Who is going to support them if I can’t? If I’m not supporting them, then they will be a burden on society forever*”.

#### 3.4.1. Exploration of Primary Support Needs

Learning and applying knowledge—Caregivers, parents, siblings and grandparents were reported to have insufficient knowledge about autism and co-occurring diagnoses, including how these conditions are characterized, experienced and impact on a young person’s functioning. Further, there was a learning need in relation to the best strategies with which to care for the young person, such as how to assist them when distressed, methods to embed therapeutic home programs into daily activities and available formal supports. Educators had learning needs regarding autism and co-occurring diagnoses, how these impact on functioning in the classroom (e.g., anxiety, motivation) and strategies to address these barriers to learning.

Communication—Caregivers described the need for collaboration between family members, health professionals, personal care providers and educators to communicate needs, establish shared goals, coordinate support plans and facilitate consistent use of strategies across settings (e.g., team meetings or other forms of communication).

General tasks and demands—Caregivers experienced challenges with complex daily routines and handling role demands and felt overloaded by the multiple responsibilities associated with supporting one or more care recipients. They reported that morning and evening routines were especially stressful, as was the need to juggle therapeutic and extracurricular activities, along with when preparing for unfamiliar situations. The physical and psychological demands of these routines were exacerbated due to the young person’s limited independence, thus requiring continuous supervision, prompting and/or assistance.

Domestic life—Many caregivers reported feeling overwhelmed by their responsibilities to assist and/or supervise the young person across multiple domains of functioning. Although these experiences were sometimes felt by other family members, caregivers frequently reported that they were primarily, or solely, responsible for supporting the young person (and occasionally other care recipients).

Services, systems and policies—Barriers included the complex landscape of services, systems and policies in Australia, spanning general social support, health and education sectors. It was difficult for caregivers to understand and navigate this landscape to ensure they could access eligible funding. In addition, it was potentially costly due to out-of-pocket expenses for supports that do not meet funding criteria or only have partial rebates available.

#### 3.4.2. Exploration of Secondary Support Needs

Mental functions—Caregivers and family members described an overwhelming sense of emotional exhaustion. They felt overloaded due to continuous supervision, multiple demands and stressful routines; overwhelmed by the young person’s impairments (in particular, those association with Mental functions); tired due to disrupted sleep routines; and/or worried about the young person.

Interpersonal interactions and relationships—Caregivers, and to some extent other family members, experienced difficulties with relationships. The extensive support needs of the young person restricted the time and emotional energy available for interaction and connection with partners, family members (in particular, their other children) and friends. This subsequently resulted in reduced intimate, family and informal relationship quality, and at times led to feeling socially isolated.

Major life areas—Some caregivers reported restricted employment participation due to caregiving responsibilities (e.g., being unable to pursue their chosen career or undertake preferred work hours). Others experienced work–life conflict due to the demands of caregiving and work. This in turn contributed to financial stress from a reduced earning capacity, exacerbated by the out-of-pocket costs of services, leading to a more complex family budget to manage.

Self-care—Maintaining their own wellbeing was challenging for caregivers. Whilst some found ways to prioritize self-care (e.g., dedicating time while the young person was at school to undertake health promoting activities), others believed they had no discretionary time available to establish self-care strategies. This resulted in inadequate rest and attention to health needs, including in some cases challenges seeking their own neurodevelopmental or mental health diagnosis and supports.

#### 3.4.3. Suggested Supports to Meet Support Needs

Support and relationships—Caregivers and family members needed access to personal care providers, health professionals and other professionals, whilst educators needed support from health professionals as part of an interprofessional team. Personal care providers were felt to play an important role in providing respite to caregivers and other family members (when support workers are spending time with the young person) so they can maintain their own health, wellbeing and employment. Health professionals, in particular psychologists, were needed to provide counselling support to caregivers and other family members. Other professionals included cleaners to assist caregivers to lessen their time demands, along with case managers to help caregivers identify and advocate for suitable supports, followed by assistance to coordinate funding and support services. Finally, health professional support was required to help caregivers, family members and educators understand the young person, their support needs and how to implement therapy programs at home and school.

Services, systems and policies—Caregivers and family members needed access to general social support services and systems, along with health and social security systems. In terms of general social support services and systems, they needed support networks, education workshops about autism and co-occurring diagnoses, respite programs and funding. Support networks provide emotional and informational support through sharing similar experiences, strategies and resources. They can be accessed through face-to-face contact or social media and be informal or formal in their group structure. Educational workshops were identified as a valuable way for caregivers, siblings and other family members to gain reliable information about autism and strategies to manage challenging situations. Respite programs, including school holiday and/or overnight programs, allow caregivers and siblings time away from caregiving, whilst providing the young person an opportunity to socialize, explore hobbies and learn life skills in a safe and supportive environment. Finally, funding for these general support services (along with counselling) was needed, and caregivers felt that the young person’s NDIS plan was usually the most appropriate source. In addition, counselling for the caregiver and other family members may be accessible through health system funding (e.g., Medicare rebates) and some caregivers may be eligible for social security system funding (e.g., income support payments).

## 4. Discussion

### 4.1. Perceived Support Needs and Suggested Supports

This study explored caregiver perspectives of support needs of 68 Australian school-aged young people on the spectrum and their support people. Overall, the perceived purpose of the support needs was to promote functioning so the young person could reach their potential as they progressed through childhood, adolescence and eventually adulthood. This is an important societal goal, as social and economic participation outcomes for autistic adults remain poor, despite significant investment in early intervention [52,53]. To meet this aim, caregivers highlighted the importance of meeting their own support needs, and those of other family members and support people, to ensure the sustainability of their caregiving capacity. This is congruent with caregiver experiences more broadly, and is an important societal goal due to the large proportion of support provided through informal support systems [54,55].

The support needs and suggested supports for the young people and their caregivers frequently spanned multiple Body Functions, Activities and Participation and/or Environmental Factors chapters of the ICF, demonstrating the complexity of challenges experienced. This is further evidenced by the hierarchical outcome spaces of interacting levels of support needs and suggested supports, and indicated that multifaceted solutions are required. There is also growing recognition that personal strengths and environmental facilitators (including informal caregivers) play an important role in mediating the impact of impairments, and should subsequently be leveraged as part of multifaceted solutions [55,56]. For the support needs that were being partially or fully met at the time of the study, caregivers highlighted the importance of ensuring the maintenance of existing informal and formal supports. In addition, almost all caregivers described supports that needed to be increased, expanded, changed or introduced. As it may not be feasible for all support needs to be fully met within the context of financial and time constraints, funders and support services should work with the young person and their caregivers to prioritize the most important support needs. Although there might be many unmet support needs, in practice neurodivergent individuals and caregivers may not have the capacity to work on more than one or two support needs concurrently [57].

The most prioritized support needs identified in this study were underpinned by the young person’s difficulties with distressing feelings, emotional regulation and/or motivation or the caregiver feeling overloaded, overwhelmed, exhausted, stressed, worried and/or conflicted (Mental functions). The primacy of this support need is supported by the representation of Mental function codes in the Brief ICF Core Set (15 of the 81 codes) [9]. Other prioritized support needs related to the young person’s challenges with school, social skills and/or friendships or the caregiver’s employment and/or relationship quality (Major life areas and Interpersonal interactions or relationships). These two chapters are represented in seven of the 81 codes in the Brief ICF Core Set [9].

When considering the full breadth of support needs identified by caregivers, six of the eight Body Functions chapters were covered to some extent. Along with Mental functions, Sensory functions and pain and Neuromusculoskeletal and movement related functions were commonly reported to under underpin the young person’s support needs. However, the former is not represented in the Brief ICF Core Set [9], despite the importance of sensory features in diagnostic criteria, and the latter is covered by only two movement related codes [9]. All nine Activities and Participation chapters were covered by the support needs. Whilst these findings are generally congruent with the Brief ICF Core Set [9], this sample of caregivers highlighted the relevance of young people developing age appropriate Domestic life skills, which is missing from the Brief ICF Core Set. Suggested supports spanned all five Environmental Factors chapters, with a focus on paid supports within Health, Disability and Education systems. The unpaid support provided by caregivers was not emphasized during the Support Needs Interview, other than in relation to threats to the sustainability of their caregiving capacity, perhaps because it was implicitly part of their core role. Interestingly, the caregivers in this sample did not raise Attitudes as a substantial issue, which is in contrast to the focus on this chapter in the ICF Core Set [9] and other research studies [54].

### 4.2. Practice Implications during the Clinical Cycle

Implications of these findings to support services, researchers and policy makers can be described in relation to a clinical cycle involving assessment, support and outcome evaluation phases [58]. During the assessment phase, support services should maintain a central focus on identifying support needs from a family-centered approach [59]. Although standardized measures of functioning (including adaptive behavior) can play a role in identifying challenges underlying support needs [16], they are unable to extrapolate if the young person and their caregiver places a high value on overcoming these difficulties and if these needs are already being met (and to what degree) by existing supports. Further, functioning and support needs measures frequently span limited domains of functioning (where environmental factors are typically underrepresented) and cannot be tailored to individual life situations [43,44,60]. Published reviews critiquing standardized measures of functioning and support needs may be helpful in determining the suitability of the measures for the clinician’s purpose, as well as potential combinations to address all required concepts [13,43,44,60]. Future research is recommended to develop and validate standardized support needs measures that are aligned with the ICF Core Sets for Autism [9]; allow active participation from the young person (self-report) and their caregivers (proxy-report); include mechanisms for individualizing the process (for example, through goal setting and other open ended questions); and can draw on existing clinical records (such as assessment of functioning results or progress reports) [16,20,61]. The authors are currently developing and piloting several measures based on the ICF Core Sets for Autism [9], and evaluation of the Support Needs Interview used in this study may also be valuable. In addition, adaptation of the Instrument for Classification and Assessment of support Needs (I-CAN) into a child and adolescent version is recommended, due to the comprehensive coverage of the full Activities and Participation domain of the ICF [8] and focus on personal goals [15]. Further, policy makers can contribute through ensuring funding decisions are made based on a rigorous assessment of the most important support needs for the individual and their family. A comprehensive clinician administered support needs assessment process may represent value for money if it leads to improved participation outcomes and unnecessary expenditure on low priority support needs.

During the support phase, support services should ensure a direct connection between assessment findings and support plans [16]. In addition, it is essential that the young person, caregiver(s) and support services agree on several support needs to prioritize at one time. Provision of supports (e.g., a recreation group) to address higher level support needs (e.g., social isolation) using a top-down approach will facilitate early engagement in personally meaningful activities (e.g., hobbies) and life situations (e.g., friendships), whilst the support service identifies and addresses lower level support needs (e.g., emotional regulation, understanding social cues, applying social rules) within naturalistic settings. These supports should also address environmental barriers (e.g., lack of personal care provider knowledge) to allow more goals to be achieved in parallel [57]. This may require innovative service planning and interprofessional collaboration to effectively embed these multifaceted elements into complex, yet engaging, supports and ensure adequate coordination for continuity and consistency [58,62]. Given the importance of evidence-based practice, researchers can assist through developing standardized supports to address higher level and complex support needs, where a template structure is used to ensure rigor, whilst still enabling flexible tailoring to individual or group needs. The could be based on a combination of peer-reviewed studies and consultation with key stakeholders (e.g., young people on the spectrum, caregivers, clinicians, other support services) [63]. This will address the current gap in the literature, where the most robust research evidence is available for supports that address lower level support needs (e.g., Mental functions, Sensory functions and pain, Communication) [64]. Policy makers may consider funding supports with an emerging evidence-base if a clear rationale is provided on how the support will address multifaceted support needs and an outcome measure is utilized [65].

During the outcome evaluation phase, support services should evaluate the effectiveness of existing and new supports that are in place to address support needs. There are several standardized outcome measures with established psychometric properties that have sufficient flexibility to be used in this context, including the Canadian Occupational Performance Measure and the Goal Attainment Scale [66,67]. Further research to evaluate standardized supports to address higher level and complex support needs is important to address the current gap in the evidence-base. This may be through collaborating with support services to analyze data from outcome measures, realist evaluations (e.g., [68,69]), inclusion as an active control intervention in a randomized control trial (e.g., [70]) and systematic/scoping reviews of emerging evidence [71]. Policy makers can facilitate this focus through embedding requirements for support services to utilize outcome measures and report on evaluation systems (at individual and service levels) and using this information to guide decision making.

### 4.3. Limitations

Whilst this study contributes a holistic exploration of the perceived support needs of school-aged young people on the spectrum and their caregivers, the findings may have been limited due to several factors. Although attempts were made to encourage caregivers with diverse gender, culturally and/or linguistically backgrounds to participate, this was only achieved to a small extent, resulting in a fairly homogenous sample of mothers. It is likely that fathers, First Nations people, migrants and non-English speaking caregivers would perceive different, and potentially additional, support needs and suggested supports. Further research could explore these perspectives specifically, facilitated through co-production, purposive sampling and participant payments [72,73]. In addition, there were multiple layers of interpretation applied during the data collection and analysis process. At the point of data collection, there were two points of interpretation—firstly the caregiver described their perceptions of the young person’s support needs and suggested supports (rather than the young person communicating these directly), and secondly the OT researcher recorded this information as written interview summaries (which were used as the primary source of data for this study). At the point of data analysis, there was a third level of interpretation when the authors completed the phenomenographic analysis process to describe the breadth and frequency of findings, which was deemed more suitable in this circumstance than seeking to understand the essence of the experiences through a phenomenological approach [34]. Subsequently, it is possible that, despite the trustworthiness strategies utilized, the perceptions of the caregivers, OT researchers and authors may have influenced the findings to some extent [57,74]. Future research could address this through recruiting young people on the spectrum to describe their support needs and suggested supports using neurodiversity-friendly data collection methods [72], utilizing interview transcripts as the primary source of data to allow a greater focus on the participants’ voices, and employing an interprofessional team of researchers to conduct the interviews.

## 5. Conclusions

The caregivers who participated in this study described a vast array of complex and interacting support needs and suggested supports related to 68 Australian school-aged young people on the spectrum and their support people. The overall goal of meeting these support needs were for the young people to optimize their functioning to reach their potential and caregivers to ensure the sustainability of their caregiving capacity. A series of recommendations for support services, researchers and policy makers have been made to positions support needs as central during the assessment, support and evaluation phases.

## Figures and Tables

**Table 1:** Description of the demographic and clinical characteristics of the young people (*n* = 68) and caregivers (*n* = 70), families and supports, reported as mean (*M*) and standard deviation (*SD*) or frequency (*n* and %).

Demographic and Clinical Characteristics	*M* (*SD*)	*n* (%)
Young People		
Age (years)	10.82 (3.09)	
Gender		
Male		42 (61.76)
Female		26 (38.24)
School Level		
Lower primary (pre-primary to Year 3)		25 (36.76)
Upper primary (year 4 to 6)		20 (29.41)
Lower secondary (year 7 to 9)		17 (25.00)
Upper secondary (year 10 to 12)		6 (8.82)
Autism Diagnosis		
Autism Spectrum Disorder		57 (83.82)
SCI Level 1		21 (38.89)
SCI Level 2		25 (46.30)
SCI Level 3		8 (14.81)
RRB Level 1		26 (48.15)
RRB Level 2		22 (40.74)
RRB Level 3		6 (11.11)
Asperger’s Syndrome		2 (2.94)
Autistic Disorder		8 (11.76)
Not Specified		1 (1.47)
Age of Initial Autism Diagnosis (years)	5.70 (2.94)	
Autistic Signs and Symptoms (AMSE ≥ 5 threshold)	5.12 (2.39)	45 (68.18)
Co-Occurring Diagnosis		
Attention Deficit/Hyperactivity Disorder		25 (36.76)
Communication Disorder		13 (19.12)
Global Developmental and/or Motor disorder		12 (17.65)
Intellectual disability		15 (22.06)
Vineland-3		
Adaptive Behavior Composite	65.55 (14.76)	
Communication	64.82 (18.60)	
Daily living skills	71.36 (20.06)	
Socialization	62.49 (17.61)	
Motor skills	79.97 (18.43)	
Maladaptive Behavior (Internalizing)	21.55 (1.61)	
Maladaptive Behavior (Externalizing)	19.36 (2.50)	
**Caregivers**		
Age (years)	42.77 (7.52)	
Gender		
Male		3 (4.29)
Female		67 (95.71)
Relationship		
Parent		66 (98.51)
Grandparent		1 (1.49)
Health and Wellbeing ^1^		
EQ-5D-5L Visual Analogue Scale	67.81 (19.39)	
Personal Wellbeing Index	59.01 (22.73)	
**Families**		
Family history of neurodevelopmental conditions		
No		31 (53.45)
Yes		27 (46.55)
Cultural Diversity		
First Nations ^2^		1 (1.52)
Belongs to a specific cultural group ^3^		13 (19.12)
Language other than English at home ^4^		8 (11.76)
State Location		
New South Wales		14 (20.90)
Queensland		7 (10.45)
Victoria		25 (37.31)
Western Australia		21 (31.34)
Geographical Location		
Major city		60 (89.55)
Inner or outer regional center		7 (10.45)

Notes. ^1^ Data available for *n* = 27. ^2^ One young person—caregiver dyad. ^3^ Six young person—caregiver dyads, two young people and five caregivers. ^4^ Three young person—caregiver dyads and five caregivers.

**Table 2:** Frequency (*n* and %) of caregivers reporting a support need and/or suggested support related to each domain and chapter of the International Classification of Functioning, Disability and Health (ICF [7]).

ICF Domain and Chapter	*n* (%)
Body Functions	66 (97.06)
b1. Mental functions ^1^	66 (97.06)
b2. Sensory functions and pain	30 (44.12)
b3. Voice and speech functions ^2^	1 (1.47)
b4. Functions of the cardiovascular, hematological, immunological and respiratory systems	6 (8.82)
b5. Functions of the digestive, metabolic and endocrine systems	5 (7.35)
b7. Neuromusculoskeletal and movement related functions	26 (38.24)
Activities and Participation	68 (100.00)
d1. Learning and applying knowledge	54 (79.41)
d2. General tasks and demands	44 (64.71)
d3. Communication	49 (72.06)
d4. Mobility	19 (27.94)
d5. Self-care	46 (67.65)
d6. Domestic life	43 (63.24)
d7. Interpersonal interactions and relationships	64 (94.12)
d8. Major life areas	61 (89.71)
d9. Community, social and civic life	54 (79.41)
Environmental Factors	68 (100.00)
e1. Products and technology	45 (66.18)
e2. Natural environment and human-made changes to environment	14 (20.59)
e3. Support and relationships	68 (100.00)
e4. Attitude	8 (11.76)
e5. Services, systems and policies	59 (86.76)

Notes. ^1^ Support needs relating to the overlapping codes of b152 Emotional functions, b164 Higher-level cognitive functions, d240 Handling stress and other psychological demands and d250 Managing one’s own behavior have been thematically coded under b1 Mental functions. ^2^ b3 Voice and speech functions was added during the inter-rater agreement process when it was identified that an interview summary had been incorrectly attributed to d3 Communication, hence it is possible that other interview summaries may mention support needs related to b3 Voice and speech functions.

**Table 3:** Examples of activities at home and in the community that the young people need support with.

Activity Category	Home	Community
Play	Backyard games Card games Electronic device games	Riding bicycle or walking in neighborhood Visiting playgrounds Swimming in public pools Play dates
Hobbies	Listening to music Making crafts Telling stories	Outing to shopping centers Watching a movie at the cinema Interest based classes (e.g., art, drama, music)
Sports	Backyard sports (e.g., shooting basketball goals)	Group lessons (e.g., ballet, gymnastics, swimming, taekwondo, yoga) Team sports (e.g., soccer, tennis) Autism or disability specific sports program
Informal associations	Not applicable	Youth clubs (e.g., Scouts) Activity based social skills groups (e.g., coding, cooking, Lego, Minecraft, theatre, writing)

## Data Availability

The data presented in this study are not publicly available as ethics approval was not obtained to share individual level data. Interested parties may contact the corresponding author to discuss access to aggregated data.

## Supplementary Materials

The following supporting information can be downloaded at: https://www.mdpi.com/article/10.3390/ijerph192315605/s1, File S1: Data collection protocol (Contains additional references: [75,76,77,78,79]).
