# Supplementary material for: Perceived Support Needs of School-Aged Young People on the Autism Spectrum and Their Caregivers
Source: Int J Environ Res Public Health. 2022 Nov 24;19(23):15605. doi: 10.3390/ijerph192315605 (PMC9737194; doi:10.3390/ijerph192315605)
Supplement: Supplementary file 1 [file ijerph-19-15605-s001.zip › ijerph-1974398-supplementary.pdf]

Perceived Support Needs of School-Aged Young People on the Autism Spectrum and their Caregivers

Kiah Evans[1-4]*, Andrew J.O. Whitehouse[1,3], Emily D'Arcy[1,3-5], Maya Hayden-Evans[1,3-5], Kerry Wallace[1], Rebecca Kuzminski[1,3-4], Rebecca Thorpe[1], Sonya Girdler[2-4,6,], Benjamin Milbourn[1,3-4], Sven Bölte[4,6-7] and Angela Chamberlain [1,3-4]

1 Telethon Kids Institute, University of Western Australia, Perth, Australia.
2 School of Allied Health, University of Western Australia, Perth, Australia.
3 Cooperative Research Centre for Living with Autism (Autism CRC), Long Pocket, Brisbane, Queensland, Australia.
4 Curtin Autism Research Group and School of Allied Health, Curtin University, Perth, Australia.
5 School of Psychological Science, University of Western Australia, Perth, Australia.
6 Karolinska Institutet Center of Neurodevelopmental Disorders (KIND), Centre for Psychiatry Research, Department of Women's and Children's Health, Karolinska Institutet, & Stockholm Health Care Services, Region Stockholm, Stockholm, Sweden.
7 Child and Adolescent Psychiatry, Stockholm Health Care Services, Region Stockholm, Stockholm, Sweden.

* Correspondence: kiah.evans@uwa.edu.au; Tel.: +61 8 6488 6894

Supplementary File - S1. Data Collection Protocol

Background Survey – Caregiver-reported

- Do you require an interpreter? [No, I can complete the assessment in English / [i]I need an interpreter who can speak (Arabic / Cantonese / Croatian / Filipino / French / German / Greek / Hindi / Indonesian / Italian / Korean / Macedonian / Mandarin / Punjabi / Serbian / Spanish / Tagalog / Turkish / Vietnamese)]
- Would you prefer to discuss this research project through a telephone call, instead of reading about the research project here? [No / Yes]

Background and Consent (Caregiver Demographic Characteristics)

Research Project Title: Reliability, Validity and Usability of Assessment of Functioning Tools for Autism Spectrum Disorder and Neurodevelopmental Conditions in the Australian Context

Principal Investigators: Dr Kiah Evans and Professor Andrew Whitehouse

Please feel free to download a copy of the Participant Information Sheet: [Link Provided]
[Consent statement and contact details]

- I agree to the above: [No / Yes]
- What is the [first and last] name of the young person within your care?
- I am a parent or caregiver to a young person [name] with ASD or a neurodevelopmental condition (aged 20 years or younger) who is currently an NDIS participant (this individual, [name], is referred to throughout this survey as 'the young person within your care'): [No / Yes]
- Do you agree to being audio and/or video recorded during the assessment? [No / Yes]
- Do you agree to video recordings being used to capture images of the young person within your care? [No / Yes]
- In addition, I give consent to having my de-identified data being use in other related studies in the future. [No / Yes]
- In addition, I agree to be contacted again regarding future research conducted by the research investigators. [No / Yes]

---

[i] This response option presented in each specified language.

- Do you need a second consent screen to be provided for another parent / caregiver to participate in the Background Survey? [No / Yes] (If yes, second caregiver completes 'Background and Consent', 'Contact Details and Availability' and 'About You' sections)

Contact Details and Availability

- What is your [first and last] name?
- What is your email address?
- What is your mobile number?
- What is your home telephone number?
- [ii]What is your postal address?
- What is your residential address?
- What [days and times] may you be available to participate in an assessment, where the young person within your care can be present for at least part of the assessment?
- Please specify any requirements or requests in relation to attending a face-to-face assessment with the young person within your care, if this were to take place at a [local clinic or your home].

About You – Demographic Characteristics

- What is your gender? [Male / Female / Other: Please feel free to describe your gender in your own words]
- What is your date of birth?
- [iii]Are you...? [An Aboriginal person / A Torres Strait Islander person / An Aboriginal and Torres Strait Islander person / None of the above]
- [iii]Do you identify as belonging to a specific cultural group? [No / Yes: Please specify]
- [iii]Do you speak a language other than English at home? [No / Yes: Please specify]
- What is the relationship with the young person within your care? [I am their biological parent / I am their step-parent / I am their adoptive parent / I am their grandparent / I am their sibling / I am their spouse / I am their friend / I am their foster carer / Other: Please specify]
- Do you live with the young person within your care at least 50% of the time? [No / Yes]
- If you complete [various activities for this project], you will entitled to a gift voucher for your family. Please select the format you prefer: [Electronic gift voucher emailed to you / Physical gift voucher posted to your home address / Donation to a charity of your choice: Please nominate]

About the Young Person Within Your Care – Demographic and Clinical Characteristics[iv]

- What is the gender of the young person within your care? [Male / Female / Other: Please feel free to describe the gender of the young person within your care in your own words]
- What is the date of birth for the young person within your care?
- [iii]Is the young person within your care...? [An Aboriginal person / A Torres Strait Islander person / An Aboriginal and Torres Strait Islander person / None of the above]
- [iii]Does the young person within your care identify as belonging to a specific cultural group? [No / Yes: Please specify]

[ii] Post code utilised to determine family state / geographical location and socio-economic status.

[iii] Combined to describe family cultural diversity.

[iv] Questions on the following topics were included in the Background Survey for the larger research project, but are not included here as they were not utilized specifically in this study (although information may have been available for file review prior to the home-visit): clinical history (developmental, medical and support), employment, other core activities, interests, skills, funding and advocacy. In addition, the following standardize measures were embedded in the Background Survey: Participation and Environment Measure for Young Children (YC-PEM) [1] or Participation and Environment Measure for Children and Youth (PEM-CY) [2], Global Assessment of Character Strengths-24 [3] and Beach Center Family Quality of Life Scale [4].

- [iii]Does the young person within your care speak a language other than English at home? [No / Yes: Please specify]
- Which condition(s) has the young person within your care been diagnosed with? (select all that apply) [Autism spectrum disorder (DSM-5) / Autistic Disorder (DSM-IV) / Asperger's Disorder (DSM-IV) / Pervasive Developmental Disorder, Not Otherwise Specified (DSM-IV) / Attention deficit/hyperactivity disorder / Communication, language and/or speech disorder / Coordination, motor and/or movement disorder / Cerebral palsy / Global developmental disorder / Intellectual disability / Learning disorder / Neurobehavioral disorder associated with prenatal alcohol exposure (FASD) / Tic disorder / Other: Please specify name of condition]
    - If Autism spectrum disorder (DSM-5) selected, please select assigned level:
        - Social communication [Level 1 (Requiring support) / Level 2 (Requiring substantial support) / Level 3 (Requiring very substantial support)]
        - Restricted, repetitive behaviors [Level 1 (Requiring support) / Level 2 (Requiring substantial support) / Level 3 (Requiring very substantial support)]
    - You have previously stated that the young person within your care has been diagnosed with the following conditions [repeat and insert name of each selected condition separately] - How old was the young person within your care when this diagnosis was given? (Please indicate in years)
- Does the young person within your care have a diagnosis of intellectual disability (e.g. DSM-5 term), mental retardation (e.g. DSM-IV term) or an IQ of <70? [No / Yes]
- Does the young person within your care experience intermittent or fluctuating levels of functioning (e.g. due to epilepsy or another co-occurring condition or symptom)? [No / Yes: Please describe]
- Is the young person within your care undergoing a diagnostic process for any other condition(s)? [No / Yes: Please specify]
- [v]Does the young person within your care have a biological relative(s) who has been diagnosed with autism or another neurodevelopmental condition? [No / Yes: Please specify who, and the condition]
- How is the young person within your care currently engaged in education? [They do not attend daycare or school yet / Daycare / childcare centre / Pre-school program / Kindergarten / Pre-primary / Year 1 / Year 2 / Year 3 / Year 4 / Year 5 / Year 6 / Year 7 / Year 8 / Year 9 / Year 10 / Year 11 / Year 12 / Apprenticeship / TAFE / Other non-university training course / University - undergraduate degree / University - postgraduate degree / They are no longer in the education system / Other: Please specify]

About Your Health and Wellbeing – Clinical Characteristics

- EQ-5D-5L [5]
- Personal Wellbeing Index-Adult [6]
- If you have any other comments, please feel free to add them below.

[v] Utilized to describe family history of neurodevelopmental condition(s).

Home-visit

Context

- Scope: 'Assessment of Functioning' as per Australia's national guideline for the assessment and diagnosis of autism [7].
- Setting: Family home at a time convenient to family.
- Present: Young person, caregiver(s) and occupational therapy researcher

Activities During Home-visit

- Build rapport.
- Confirm / obtain consent and assent (as applicable).
- Administer Pediatric Evaluation of Disability Inventory Computer Adaptive Test [8].
- Collect information to complete International Classification of Functioning, Disability and Health (ICF) Core Sets for Autism Documentation Form[vi]:
    - Card sort interview with caregiver(s); and
    - Interaction with / observation of young person.
- Administer Autism Mental Status Exam [11].
- Conduct Support Needs Interview (Figure 1).

**SUPPORT NEED A**<br>Short name:	
Please describe the unmet need:	
Please describe any supports that are already in place (include detail who, how often and the intensity of the support):<br>*E.g. Jack currently receives help from his mother (SOURCE) to get dressed (TYPE) every morning (FREQUENCY), this usually is usually through reminders and checking task completion (INTENSITY) but some times more help is provided, such as if there are complicated buttons, shoelaces or if Jack is in a distressed mood (INTENSITY).*	
Please describe any new supports that the parent/caregiver or young person has identified as required (include detail who, how often and the intensity of the support):	
Please describe any new supports that the assessor has identified as required (include detail who, how often and the intensity of the support):	
Other comments about support need:	
**REPEAT FOR UP TO 10 SUPPORT NEEDS**<br><br>**RANK SUPPORT NEEDS FROM MOST (1) TO LEAST (≤10)**	

Figure 1. Support Needs Interview.

[vi] Developed by the research team based on the following resources: [8, 9]. Please contact PhD students Emily D'Arcy emily.darcy@postgrad.curtin.edu.au and Maya Hayden-Evans maya.hayden-evans@postgrad.curtin.edu.au for more details.

Activities After Home-visit

- Administer Vineland Adaptive Behavior Scales, Third Edition Comprehensive Interview via telephone[vii] [12].
- Prepare Assessment of Functioning Report using information collected from Background Survey and Home-visit (including activities during and after).
- Share Assessment of Functioning Report with caregiver(s).
- Member checking and feedback session with caregiver (telephone, video conference or survey).

References

1. Khetani, M.; Coster, W.; Law, M.; Bedell, G., *Young Children's Partcipation and Environment Measure (YC-PEM): User guide*. CanChild, McMaster University: Hamilton, Canada, 2013.
2. Coster, W.; Law, M.; Bedell, G., *Partcipation and Environment Measure for Children and Youth (PEM - CY): Form & user ' s guide*. CanChild, McMaster University: Hamilton, Canada, 2010.
3. McGrath, R., *The VIA assessment suite for adults: Development and initial evaluation - Technical report*. VIA Institute on Character: Cincinnati, United States, 2019.
4. Beach Center on Disability, *Beach Center Family Quality of Life Scale: Psychometric characteristics and scoring key*. University of Kansas: Lawrence, United States, 2015.
5. EuroQol Research Foundation, *EQ-5D-5L user guide (version 3)*. EuroQol Research Foundation: Rotterdam, The Netherlands, 2019.
6. The International Wellbeing Group, *Personal Wellbeing Index – Adult (English) manual*. 5th ed.; Centre on Quality of Life, Deakin University: Melbourne, Australia, 2013.
7. Whitehouse, A.; Evans, K.; Eapen, V.; Wray, J., *A national guideline for the assessment and diagnosis of autism spectrum disorders in Australia*. Autism CRC: Brisbane, Australia, 2018.
8. Haley, S.; Coster, W.; Dumas, H.; Fragala-Pinkham, M.; Moed, R.; Kramer, J.; Ni, P.; Feng, T.; Kao, Y.; Ludlow, L., *Pediatric Evaluation of Disability Inventory: Development, standardization and administration manual (Version 1.4.3)*. Boston University: Boston, United States, 2019.
9. Bölte, S.; Mahdi, S.; de Vries, P.; Granlund, M.; Robison, J.; Shulman, C.; Swedo, S.; Tonge, B.; Wong, V.; Zwaigenbaum, L., The gestalt of functioning in autism spectrum disorder: Results of the international conference to develop final consensus International Classification of Functioning, Disability and Health core sets. *Autism* **2019,** 23, (2), 449-467, 10.1177/1362361318755522.
10. World Health Organization, *International Classification of Functioning, Disability, and Health*. World Health Organization: Geneva, Switzerland, 2001.
11. Grodberg, D.; Weinger, P.; Kolevzon, A.; Soorya, L.; Buxbaum, J., Brief report: The Autism Mental Status Examination: Development of a brief autism-focused exam. *Journal of Autism and Developmental Disorders* **2012,** 42, (3), 455-459, 10.1007/s10803-011-1255-4.
12. Sparrow, S.; Cicchetti, D.; Saulnier, C., *Vineland Adaptive Behaviour Scales, Third Edition*. Pearson Inc: 2016.

[vii] May administer prior to home-visit to accommodate family's schedule.
